# Understanding and Addressing Barriers to Care for LGBTQ+ Older Adults Living with Subjective Cognitive Problems

**DOI:** 10.1093/geroni/igaf122.332

**Published:** 2025-12-31

**Authors:** Jessica VanderWerf, Sarah McCrackin, Nadine Austin, Nik Lampe

**Affiliations:** University of South Florida, Tampa, Florida, United States; University of South Florida Health, Tampa, Florida, United States; Georgia State University, Atlanta, Georgia, United States; University of South Florida, Tampa, Florida, United States

## Abstract

Lesbian, gay, bisexual, transgender, and queer (LGBTQ+) older adults living with cognitive health issues face multiple, compounding barriers in accessing and utilizing quality care. These barriers are driven by structural mechanisms such as providers lacking LGBTQ+ medical education and training, limited culturally tailored resources, and the exacerbated effects of the family caregiver availability crisis due to policies restricting access to marriage and family formation. In this study, we aim to answer the following research question: “What are the unique or specific barriers to healthcare services, support, and resources for LGBTQ+ older adults living with subjective cognitive problems?” From June 2024 to February 2025, the study team conducted semi-structured interviews (N = 22) with LGBTQ+ adults aged 60+ diagnosed with mild cognitive impairment (MCI) by a licensed clinician (n = 8); or who self-reported significant cognitive health problems (n = 9); and licensed clinicians (n = 5). Data were coded using NVivo software and analyzed inductively through grounded theory analysis. Three thematic themes were analyzed: (1) lack of LGBTQ+ culturally-responsive medical education and training; (2) comorbidities, including social isolation, depression, and anxiety, that complicate barriers to care; and (3) systemic biases and facility-related issues on LGBTQ+ patients in cognitive care settings. These findings highlight the need to enhance and expand culturally tailored programs, services, support, and resources for LGBTQ+ older populations living with cognitive health issues and conditions. This study highlights the need for culturally tailored programming, improved care access, and LGBTQ+ inclusive policy initiatives to address the unique barriers faced by this medically vulnerable population.

